# Hippocampal non-theta state: The “Janus face” of information processing

**DOI:** 10.3389/fncir.2023.1134705

**Published:** 2023-03-07

**Authors:** Ivan Mysin, Liubov Shubina

**Affiliations:** Laboratory of Systemic Organization of Neurons, Institute of Theoretical and Experimental Biophysics of Russian Academy of Sciences, Pushchino, Russia

**Keywords:** delta rhythm, large irregular activity, medial septum, slow wave sleep, local field potential, pyramidal neurons, interneurons, quiet wakefulness

## Abstract

The vast majority of studies on hippocampal rhythms have been conducted on animals or humans in situations where their attention was focused on external stimuli or solving cognitive tasks. These studies formed the basis for the idea that rhythmical activity coordinates the work of neurons during information processing. However, at rest, when attention is not directed to external stimuli, brain rhythms do not disappear, although the parameters of oscillatory activity change. What is the functional load of rhythmical activity at rest? Hippocampal oscillatory activity during rest is called the non-theta state, as opposed to the theta state, a characteristic activity during active behavior. We dedicate our review to discussing the present state of the art in the research of the non-theta state. The key provisions of the review are as follows: (1) the non-theta state has its own characteristics of oscillatory and neuronal activity; (2) hippocampal non-theta state is possibly caused and maintained by change of rhythmicity of medial septal input under the influence of raphe nuclei; (3) there is no consensus in the literature about cognitive functions of the non-theta-non-ripple state; and (4) the antagonistic relationship between theta and delta rhythms observed in rodents is not always observed in humans. Most attention is paid to the non-theta-non-ripple state, since this aspect of hippocampal activity has not been investigated properly and discussed in reviews.

## 1. Introduction

Information procession requires a dynamic coordination of activity of large groups of neurons in different brain structures. Brain rhythms organize neuronal activity in sensory information processing and cognitive tasks. The coordination of neurons is necessary both at the level of information transmission between brain structures and the neuronal ensembles within one brain area (Fries, 2015).

Rhythmic activity in the brain does not disappear during rest, when attention is not focused on external stimuli. But does it carry an information load or is it an “offline” state for neuronal computations? What are the mechanisms of transition between different rhythmic modes during active behavior and the resting state? We will consider these questions in regard to the hippocampus, since a lot of information about rhythms was obtained on this brain structure. The case of patient HM demonstrated the impossibility of acquisition of new memories without the participation of the hippocampus (Corkin, 2002; Buzsáki and Moser, 2013). The discovery of place cells, hippocampal neurons encoding the space, made the hippocampus a model object of memory research at the neuronal level (O’Keefe, 1976). The investigation of attention shows that the hippocampus is a central link in detecting the novelty of stimuli. It is probably the hippocampus that determines which information is new and requires memorization (Vinogradova, 2001; Numan, 2015).

There are two main functional states or modes of the hippocampal activity, theta and non-theta states (Buzsáki, 2002; Schultheiss et al., 2020). They have different behavioral correlates and a clearly different spectral content of local field potentials (LFPs) and neuronal spiking. The hippocampal theta state manifests itself during active exploratory behavior, locomotion, cognitive situations requiring attention, and rapid eye movement (REM) sleep. The slow-wave sleep (SWS) and quiet wakefulness (immobility, eating, grooming) represent the non-theta hippocampal state (Vanderwolf, 1969; Buzsáki, 2002; Young and McNaughton, 2009; Colgin, 2013). In the theta state, hippocampal LFPs exhibit strong regular theta (4–12 Hz) and gamma (30–120 Hz) oscillations (Bragin et al., 1995; Csicsvari et al., 2003; Tort et al., 2009; Belluscio et al., 2012). In the non-theta state, hippocampal activity is dominated by slower and often irregular oscillations in the delta (0.5–4 Hz) frequency range, interrupted by highly synchronous neuronal activity, sharp wave-ripple complexes (SWRs; Buzsáki, 2015). Theta and non-theta hippocampal states are traditionally characterized as two opposite and mutually exclusive states, the “online” and “offline” modes of information processing (Buzsáki, 2002).

However, “offline” does not mean that there is no active information processing. In the non-theta state, it was shown that memory consolidation takes place (Carr et al., 2011; Vandecasteele et al., 2014; Buzsáki, 2015; Mizuseki and Miyawaki, 2017). Moreover, recent studies demonstrated that in this mode, information is transferred from the hippocampus to other brain structures. For example, it was found that the activation of neuronal ensembles duri SWS in the medial prefrontal cortex (PFC) correlates with the preceding hippocampal ripples (Todorova and Zugaro, 2020).

The research on hippocampal rhythms is mainly devoted to the theta state. From numerous studies and reviews (Vinogradova, 1995; Buzsáki, 2002; Colgin, 2013; Tsanov, 2015), we know about the theta-gamma coupling (Belluscio et al., 2012), phase precession of place cells (O’Keefe and Recce, 1993), theta and gamma coherence between hippocampal regions during cognitive tasks, etc. (Mysin and Shubina, 2022). The non-theta state is investigated principally in the context of ripple oscillations (Buzsáki, 2015). Other aspects, such as delta and gamma rhythms, the functional significance of the non-theta mode and the transition between operational modes and its possible mechanisms, are poorly known and have not yet been discussed in reviews. We will try to correct this “bias” and systematize the available experimental data concerning the hippocampal non-theta state. We propose possible mechanisms underlying the transition between hippocampal theta and non-theta states and review the currently available data on the functional significance of the non-theta mode. We focus mainly on studies of natural waking and sleep states and studies on anesthetized animals are specifically mentioned.

## 2. Hippocampal activity in the non-theta state

**Figure 1:**
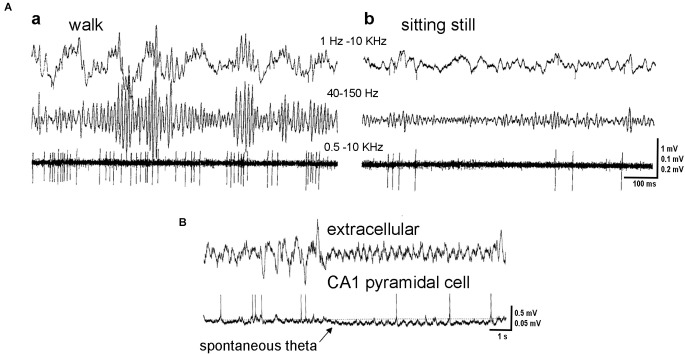
Hippocampal activity in theta and non-theta states. **(A)** Activity in the hilar region. Microelectrode recording during exploratory walking **(a)** and sitting motionless **(b)**. Upper trace, wide band recording. Middle and lower traces, gamma activity (40–150 Hz) and unit firing (500 Hz to 5 kHz), respectively. Note theta- and gamma-related modulation of the isolated neuron. **(B)** Simultaneous recording of local field potentials in the CA1 pyramidal layer (extracellular) and intracellular activity of an identified pyramidal cell. Note hyperpolarization of the pyramidal cell membrane at the onset of spontaneous theta activity (arrow). Adapted with permission from Bragin et al. (1995) **(A)**, Freund and Buzsáki (1996) **(B)**.

Hippocampal network activity is spatially and temporally tuned in a behavior-dependent manner. During quiet wakefulness, consummatory behavior and grooming non-theta activity replaces “theta state” patterns (Figure 1). This activity includes low-frequency oscillations of different amplitudes, SWRs, and SWRs-associated gamma rhythms (Buzsáki et al., 1983; Buzsáki, 1986; Bragin et al., 1992, 1995). It should be noted that similar electrophysiological activity could be registered in the hippocampus during drowsiness and SWS (Jarosiewicz et al., 2002; Buzsáki, 2015).

**Figure 2:**
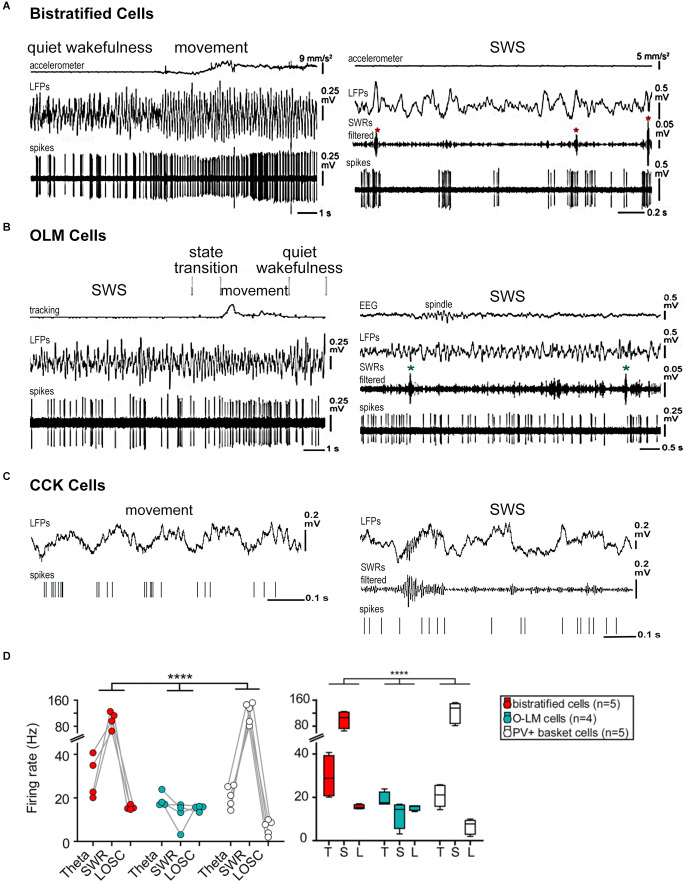
Firing patterns of hippocampal interneurons during different functional modes. **(A)** Behavior-related activity of bistratified cells. The bistratified cell increased its firing rate and changed to a more regular rhythmic pattern at a transition from quiet wakefulness to movement. Note the SWR-related (asterisks) strong increases in the firing rate. **(B)** Activity of OLM cells related to behavior. The rhythmic firing of the OLM cell during movement became irregular during SWS. The cell did not appear to change its activity during SWRs (asterisks). **(C)** Firing sequences of an interneuron (presumably CCK cell) during theta (movement) and SWRs oscillations (during SWS) recorded in the CA1 pyramidal layer. The cell fired at the ascending phase of the theta cycle and at the beginning and end of the ripple episode. **(D)** Bistratified, OLM, and PV basket cell activity during network oscillations. *Left*: different mean firing rates during different network oscillatory states (*p* < 0.0001 for the interaction; repeated-measures ANOVA). *Right*: box plot of cell-type-specific and oscillatory state-dependent firing rates. Abbreviations: CCK, cholecystokinin-containing cells; LFPs, local field potentials; L, LOSC, low oscillatory periods; OLM, oriens lacunosum moleculare cells; PV, parvalbumin-expressing cells; SWRs, sharp wave-ripple oscillations, SWS, slow-wave sleep; T, theta oscillations. Adapted with permission from Klausberger et al. (2005) **(C)**, Katona et al. (2014) **(A,B,D)**. **** means high statistical significance (*p* < 0.0001).

The functional state of the brain modulates the firing rate and patterns of individual neurons in the hippocampus (Figures 1, 2). In 1987 Colom and Bland described, in urethane-anesthetized rats, the presence in the hippocampus of “theta-off” cells, which differed from “theta-on” classical “theta” cells. “Theta-off” cells generated non-complex-spikes and were inactive during theta activity, discharging preferentially during the non-theta state (Colom and Bland, 1987; Ford et al., 1989). Later, in addition to theta-related cells, theta-unrelated neurons were recorded (also in urethane-anesthetized animals), which discharged with simple or complex spikes or were even “silent”. Thus, hippocampal pyramidal neurons were functionally heterogeneous in the generation of current LFPs (Bland et al., 2005).

The difference between hippocampal operational modes (theta and non-theta states) is also manifested in pathological conditions such as seizure activity. It was shown in a rat model of temporal lobe epilepsy that the hippocampal functional state preceding seizures influenced spontaneous seizure activity and determined the success of therapeutic intervention. Optogenetic stimulation significantly curtails seizures that emerged from non-theta states (Ewell et al., 2015).

In this section we summarize data on hippocampal LFPs and neuronal activity in the non-theta state. We will start with less popular low-frequency oscillations and then move on to much more studied SWRs.

### 2.1 Low-frequency oscillations

#### 2.1.1 Large-amplitude irregular activity and delta rhythm

**Figure 3:**
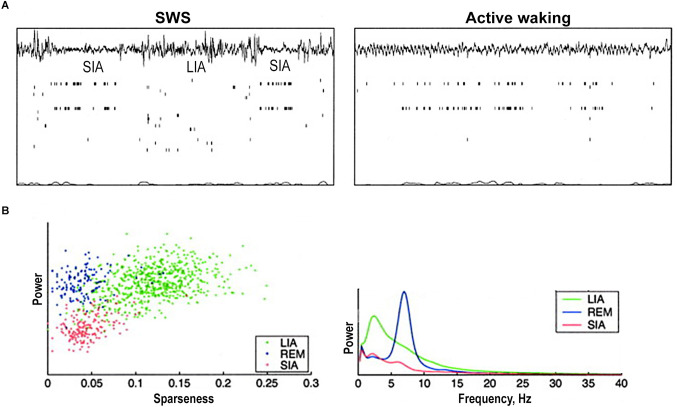
Hippocampal population activity during small irregular activity (SIA). **(A)** Two short sleep-SIA episodes within LIA *(left)*. Note the flattening of the EEG and the characteristic population activity. Later in the recording *(right)*, the rat is awake and moving around inside the nest. Note that the same subset of cells that was active in SIA is active here. **(B)** The population activity differences between sleep-SIA and the other hand-delineated sleep states. “Sparseness” of population activity is 1 when all cells are active at the same firing rate and approaches 0 when a very small fraction of cells are active and the rest are silent. Scatterplot of sparseness vs. EEG total power exhibits a robust clustering corresponding to periods of LIA, REM, and SIA: LIA has high EEG power and high sparseness, REM has high EEG power and low sparseness, and SIA has low EEG power and low sparseness. Power spectra exhibit that SIA has a lower power across the frequency spectrum than either LIA or REM. It has a small peak in the low-frequency theta range (6 Hz). REM shows a peak at theta frequency (7 Hz), and LIA has a wide peak at 2–4 Hz and remains higher than REM and SIA across the spectrum. Abbreviations: LIA, large irregular activity; REM, rapid eye movement sleep; SIA, small irregular activity; SWS, slow-wave sleep. Adapted from Jarosiewicz et al. (2002).

LFPs recorded during SWS and quiet wakefulness are often characterized as large-amplitude irregular activity (LIA). LIA is traditionally thought to be a deactivated state of hippocampal activity (Vanderwolf, 1969; Leung et al., 1982). In general, LIA represents a broadband signal with complexes of sharp waves and ripples (Buzsáki, 1986; Figure 3B). As far as the regular activity in the non-theta states is considered, one of the predominant types is delta oscillations (1–4 Hz). It was shown that delta power was maximal in non-theta states, and minimal in theta states (Young and McNaughton, 2009). In a recent study, periods of delta-dominated activity were registered in the hippocampus of freely behaving rats when animals were stationary or were moving slowly (Schultheiss et al., 2020).

The neuronal spiking in hippocampal non-theta-non-SWRs states is rarely analyzed in detail. It was shown in anesthetized rats that there is no significant difference in the discharge frequency of pyramidal cells between theta and non-theta-non-SWRs states (Klausberger et al., 2003); however, between different cells there is a large variability in this parameter in freely moving rats (Lapray et al., 2012). Pyramidal cells fire with similar mean rates during locomotion, quiet wakefulness, and SWS (Lapray et al., 2012).

At the same time, hippocampal interneurons of different types, which provide temporally and spatially distinct inhibition of specific pyramidal subcellular compartments, change their activity in different behavioral and network states (Klausberger et al., 2003; Somogyi and Klausberger, 2005; Lapray et al., 2012; Varga et al., 2012; Somogyi et al., 2014). The firing rate of most hippocampal interneurons decreases in non-theta-non-SWRs periods (Bragin et al., 1995; Czurkó et al., 2011; Varga et al., 2012; Figure 2, Table 1). In anesthetized rats, cholecystokinin-expressing (CCK) interneurons reduce their firing by almost eight times in non-theta-non-SWRs periods (Klausberger et al., 2005; Figure 2C). In quiescent rats, a decrease in the firing rate of perisomatically projecting parvalbumin-expressing (PV) basket cells (Lapray et al., 2012; Varga et al., 2012; Katona et al., 2014; Figure 2D), somatostatin-expressing distal dendritically projecting oriens lacunosum moleculare (OLM) cells (Varga et al., 2012; Katona et al., 2014; Figures 2B,D), axo-axonal interneurons (Dudok et al., 2021), and proximal dendritically projecting bistratified cells (Figures 2A,D) was observed in these periods (Varga et al., 2012; Katona et al., 2014). In contrast, the firing rate of ivy cells, which provide fine and dense projections to pyramidal cell dendrites and represent one of the most numerous hippocampal GABAergic neuronal types, remains almost unchanged during movement, sleep, and quiet wakefulness (Lapray et al., 2012).

**Table 1:** Neuronal activity during different brain states.

		Brain state	
	Theta	Non-theta	
		SWRs	Non-theta-non-SWRs
Cell Type			
*Hippocampus*			
Pyramidal cells	- Population activity is generally diffuse (Buzsáki et al., 1992; Lapray et al., 2012) - Firing rate is similar in distinct brain states; there is a large variability in the activity of different cells (Lapray et al., 2012)	- Significant peaks of synchronous neuronal activity may be observed (Buzsáki et al., 1992; Lapray et al., 2012) - Firing rate is increased (Tukker et al., 2013)	- Discharge frequency has no significant difference from theta state (Klausberger et al., 2003) - Small subset of cells become active during SIA (Jarosiewicz et al., 2002)
	- Pyramidal cell-interneuron pairs have stronger local relationships (Hirase et al., 2001)	- Synchrony of cell pairs is significantly higher, negatively correlated neuron pairs are practically absent (Mizuseki and Buzsaki, 2014) - Cross-correlation between local pairs is significantly stronger than between distant ones (Hirase et al., 2001)	
Interneurons	Activity is changed during different behavioral and network states (Klausberger et al., 2003; Somogyi and Klausberger, 2005; Lapray et al., 2012; Varga et al., 2012; Somogyi et al., 2014)		Firing rate of most interneurons is decreased (Bragin et al., 1995; Czurkó et al., 2011; Varga et al., 2012).
PV basket cells	Firing rate is changed dynamically according to the ongoing brain state (Lapray et al., 2012)	- Cells are active and significantly increase their firing rates (Klausberger et al., 2003; Varga et al., 2012; Tukker et al., 2013; Katona et al., 2014)	Firing rate is decreased (Varga et al., 2012; Lapray et al., 2012; Katona et al., 2014)
*OLM cells*		- Cells are not very active (Klausberger et al., 2003; Varga et al., 2012; Katona et al., 2014) - Cells discharge in about half of the SWRs episodes and significantly later than PVB cells - Firing probability is low, but mean firing rate increases and spiking is robustly phase locked to the SWRs (Varga et al., 2012)	Firing rate is decreased (Varga et al., 2012; Katona et al., 2014)
Axo-axonal interneurons		- Activity is suppressed (Klausberger et al., 2003; Viney et al., 2013)	Firing rate is decreased (Dudok et al., 2021)
Bistratified cells		- Firing is increased, neurons discharge in-phase with basket cells (Klausberger et al., 2004)	Firing rate is decreased (Varga et al., 2012; Katona et al., 2014)
Ivy cells		Cells often remain silent, their firing frequencies may slightly increase (Lapray et al., 2012)	Firing rate is preserved during different behavioral states (Lapray et al., 2012)
CCK cells		Cells do not exhibit a strong increase or inhibition of firing probability (Klausberger et al., 2005)	Firing rate is significantly reduced in anesthetized rats (Klausberger et al., 2005)
MS	Stimulation of the MS induces a stable theta rhythm (Brazhnik et al., 1985; Vandecasteele et al., 2014; Astasheva et al., 2015)	60% of cells are significantly suppressed (Dragoi et al., 1999)	- Inactivation of the MS or complete suppression of its neuronal activity leads to a pronounced non-theta state in the hippocampus (Green and Arduini, 1954; Petsche and Stumpf, 1962; Mitchell et al., 1982; Mizumori et al., 1990; Smythe et al., 1991; Vinogradova et al., 1993; Lawson and Bland, 1993; Wang et al., 2015)
Glutamatergic cells	Intraseptal connections are important in hippocampal theta rhythm generation (Fuhrmann et al., 2015; Robinson et al., 2016)		
Cholinergic cells	- Intraseptal connections are important in hippocampal theta rhythm generation (Vandecasteele et al., 2014; Dannenberg et al., 2015). - Cells are highly active (Ma et al., 2020)	Optogenetic activation of cholinergic cells suppresses ripple oscillations (Ma et al., 2020)	Cells are almost silent during SWS (Ma et al., 2020)
PV GABAergic cells	Activity correlates most strongly with hippocampal theta rhythm (Borhegyi et al., 2004; Varga et al., 2008; Hangya et al., 2009)		- Stimulation of both cell bodies and hippocampal terminals impedes the non-theta activity (Dannenberg et al., 2015; Zutshi et al., 2018) - Activity has no significant difference between brain states (Simon et al., 2006)
*Teevra cells*	Cells are rhythmic and fire with short bursts preferentially at the troughs of hippocampal LFPs (Joshi et al., 2017)	- Firing is not changed (Joshi et al., 2017) - Around 40% of cells are activated (Viney et al., 2013)	Cells maintain their firing rate, but decrease their rhythmicity (Joshi et al., 2017)
*Komal cells*	Cells have long burst duration preferentially at the peaks of CA1 theta LFPs (Joshi et al., 2017)		Cells reduce their firing rate (Joshi et al., 2017; Unal et al., 2018)
MRn	Suppression of MRn activity increases the frequency and regularity of discharge of theta-bursting neurons of MS and hippocampus and produced continuous theta in the hippocampus (Kitchigina et al., 1999; Vinogradova et al., 1999)		- Low-amplitude stimulation disrupted MS bursting and abolished theta activity in the hippocampus (Kitchigina et al., 1999; Vinogradova et al., 1999) - High-amplitude MRn stimulation provokes SIA, suppresses the activity of the majority of both theta-on and theta-off cells (Jackson et al., 2008)
Serotonergic neurons	- Discharging of cells disrupts theta activity of MS and/or hippocampal neurons (Vertes, 2010) - Serotonin reuptake inhibitor significantly decreases hippocampal theta rhythm (Kudina et al., 2004)		
Glutamatergic cells	Non-serotonergic (possibly glutamatergic) projections of the MRn to the MS may contribute to generation of theta state (Crooks et al., 2012)		Elevated extracellular level of glutamate (Varga et al., 1998)

Note that neuronal activity in the theta state is not detailed and is only given in relation to differences from the non-theta states.

#### 2.1.2 Small-amplitude irregular activity

Small-amplitude irregular activity (SIA) is also described in the hippocampal non-theta state (Vanderwolf, 1971; Whishaw, 1972; O’Keefe and Nadel, 1979; Jarosiewicz et al., 2002). In earlier studies on rats, SIA was observed during the abrupt cessation of ongoing movement or a sudden transition from rest or sleep to an alert state without movement (Vanderwolf, 1971; Whishaw, 1972; O’Keefe and Nadel, 1979). SIA repeated within SWS and took around 30% of SWS (Jarosiewicz et al., 2002). It was noticed that SIA occurred immediately after every REM episode, but almost never before it. LFPs during SIA were very low in amplitude, but a small subset of complex-spike cells (presumably pyramidal cells) became active (Figure 3). It was found that many of these cells have place fields corresponding to the location of the rat’s sleep (Jarosiewicz et al., 2002; Wolansky et al., 2006). Although SIA during sleep is similar to SIA during wakefulness, there is no clear evidence yet that these activities represent the same physiological state.

Some authors also distinguish large-amplitude slow-frequency oscillations (≤1 Hz) as a particular state in the hippocampal non-theta mode (Wolansky et al., 2006). This activity occurs in situations similar to the neocortical slow oscillations: natural sleep and anesthesia. However, hippocampal slow oscillations demonstrate some independence in initiation, coordination, and coherence. Sensory stimulations and cholinergic agonists abolish this activity, while increasing anesthetic depth and muscarinic antagonists enhance it (Wolansky et al., 2006).

It is not completely clear if the SIA and slow oscillations in the hippocampus are comparable in prevalence and functional significance with the LIA and theta states, but they may provide favorable conditions for synchronization-dependent synaptic plasticity and hippocampal-dependent memory consolidation.

### 2.2 High-frequency oscillations

#### 2.2.1 SWRs

Another characteristic pattern of hippocampal activity in the non-theta state is SWRs. SWRs occur periodically, 0.5–2 times per second and last 30–120 ms (Buzsáki, 1986, 2015). SWRs include large-amplitude, “sharp-wave” voltage deflections with simultaneously occurring 120–230 Hz “ripples”. A large amount of data has been accumulated about SWRs. The ideas about the mechanisms and functions of SWRs have been already well systematized in several reviews (Buzsáki, 2015; Colgin, 2016; Todorova and Zugaro, 2020). Therefore, we confine ourselves to basic data on SWRs.

As it was mentioned earlier, pyramidal cells increase their firing rates during CA1 SWRs in anesthetized (Tukker et al., 2013) and freely behaving animals (Buzsáki, 1986; Buzsáki et al., 1996). Most CA1 population bursts that occur during immobility are associated with SWRs (Malvache et al., 2016). Synchronous calcium transients registered by two-photon calcium imaging of awake head-restrained mice are often coupled with SWRs (Malvache et al., 2016).

Hippocampal interneurons demonstrate functional heterogeneity during SWRs, affecting the temporal organization of the activity of pyramidal cell ensembles (Table 1). PV basket cells both in anesthetized and freely-behaving animals are activated, and their firing rate increases (Klausberger et al., 2003; Varga et al., 2012; Tukker et al., 2013; Katona et al., 2014; Figure 2D). Bistratified cells also increase their firing, and discharge in-phase with basket cells in anesthetized rats (Klausberger et al., 2004; Figures 2A,D). In contrast, OLM cells during SWRs are not very active (Klausberger et al., 2003; Varga et al., 2012; Katona et al., 2014; Figures 2B,D). Whereas PV basket neurons fire during most SWRs, OLM cells discharge only in about half of the SWRs episodes and significantly later than PV basket cells. Even though the firing probability of OLM neurons during SWRs is low, their mean firing rate increases and their spiking is also robustly phase-locked to SWRs (Varga et al., 2012). Ivy cells also often remain silent during SWRs but in general their firing frequencies are similar during theta and low oscillatory periods, and slightly increase during SWRs although insignificantly (Lapray et al., 2012). Another cell type, suppressed during SWRs in anesthetized and freely moving rats, is axo-axonic interneurons (Klausberger et al., 2003; Viney et al., 2013). Firing pattern of CCK interneurons differs from the other interneurons (Klausberger et al., 2005). This type of neurons, on average, has no correlation to SWRs (Figure 2C). The same CCK cell may be sometimes suppressed and sometimes excited during SWR episodes in anesthetized animals (Klausberger et al., 2005).

Thus, the suppression of one type of GABAergic neurons and the activation of the others redistribute inhibition in pyramidal cells at the subcellular level, creating conditions for the initiation of SWRs and may dynamically regulate network computations in different behavioral states.

The data on the activity of neurons are important for describing the mechanisms of SWRs. There are several theoretical models that explain the generation of SWRs and neuronal activity during these oscillations. The central idea of many theoretical works is that SWRs arise as a result of the propagation of excitation in the form of a traveling wave between pyramidal neurons of one neural ensemble (Taxidis et al., 2012; Omura et al., 2015; Malerba et al., 2016; Aussel et al., 2018). These models reproduce well the activation profile of pyramidal neurons and a pattern of LFPs during SWRs. Other hypotheses also tried to explain neuronal synchronization during SWRs. Thus, Traub and Bibbig (2000) attributed the generation of SWRs to the presence of gap junctions between pyramidal neurons. Holzbecher and Kempter (2018) suggested that PV basket neurons synchronize their activity through gap junctions and transmit a synchronous signal to pyramidal cells.

As a trigger of hippocampal SWRs may serve another brain structure, presumably, the entorhinal cortex (Sirota et al., 2003; Isomura et al., 2006). It was found that acute optogenetic inactivation of the medial entorhinal cortex layer III during quiet wakefulness reduced the incidence rate of hippocampal SWRs (Yamamoto and Tonegawa, 2017). But on the other hand, recent analysis of hippocampal activity in the resting state did not show any increase or decrease in the rate of place cell bursts or pattern co-activation rates in animals with bilateral excitotoxic lesions of the medial entorhinal cortex (Chenani et al., 2019).

It is not quite clear whether the CA3 and CA1 SWRs are generated independently, or they are interrelated. Simultaneous recordings of neuronal ensembles in behaving rats demonstrated that CA3 population bursts may provoke the depolarization of the apical dendrites of CA1 pyramidal cells, and, jointly with the excitation of certain types of CA1 interneurons, create conditions for the generation of CA1 SWRs. Moreover, the location of CA1 SWRs can be predicted by the activity of single CA3 pyramidal neurons (Csicsvari et al., 2000). However, the coupling of CA3 basket cells only to local SWRs and not to CA1 SWRs in anesthetized animals suggests the possibility of independent generation of fast oscillations in CA1 and CA3 (Tukker et al., 2013).

Summarizing, in non-theta states, the population activity of hippocampal pyramidal cells is generally irregular and scattered (Figure 1B) but it increases largely during SWRs (Buzsáki et al., 1992; Lapray et al., 2012) when significant peaks of synchronous neuronal activity are observed. Hippocampal interneurons of different types change their activity according to the ongoing brain state in different ways (Klausberger et al., 2003; Klausberger and Somogyi, 2008; Figure 2). Interneurons may coordinate the activity of pyramidal cells in a brain-state-dependent manner by innervating particular domains of pyramidal cells.

#### 2.2.2 SWRs-associated gamma

SWRs may be accompanied by slow and fast gamma not only during active behavior, but also in non-cognitive states (Carr et al., 2012). However, during immobility, consummatory behavior, drowsiness, and SWS, the amplitude of gamma is much lower than in the theta-state (Leung et al., 1982; Buzsáki et al., 1983; Bullock et al., 1990; Bragin et al., 1995).

**Figure 4:**
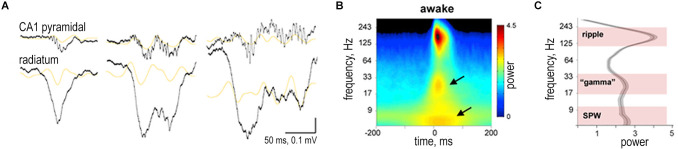
Slow gamma during sharp wave-ripple complexes (SWRs). **(A)** Examples of different length SWRs. Wide-band (1 Hz to 20 kHz) LFP traces from the CA1 pyramidal layer and *str. radiatum*. Orange traces are 20–50 Hz filtered LFPs. Gray traces are power envelope in the 100–300 Hz band. **(B)** Average peri-SWR wavelet spectrogram (*n* = 12 animals) during waking immobility. In addition to the ripple (150 Hz), two other spectral components appear in lower-frequency bands, one around 30 Hz and other below 10 Hz (arrows). **(C)** Derivative of peri-SWR wavelet spectrogram at the SWR peak from 5–300 Hz identifies three spectral peaks: 120–250 Hz (ripple band), 17–40 Hz (gamma band), and 5–15 Hz (sharp-wave band). Reproduced with permission from Oliva et al. (2018).

It was demonstrated in rats that, during SWRs in both W-track run sessions and interleaved rest sessions, the amplitude of slow gamma (20–50 Hz) transiently increased (Carr et al., 2012). The converse was also true: gamma power in CA1 was predictive of the presence of SWRs; the probability of concurrent SWRs increased with increasing CA1 gamma power. SWRs and the slow gamma exhibited strong cross-frequency coupling: the amplitude of CA1 SWRs peaked during the early descending phase of the slow gamma (Carr et al., 2012; Figure 4). During SWRs in both active and quiescent states, CA3-CA1 slow gamma coherence also increased, and the spiking in CA1 and CA3 was phase-locked to gamma oscillations. Such gamma modulation and the increase in slow gamma synchrony during SWRs in quiescence were always less pronounced than during active states. The described effects were characteristic of both non-theta wakefulness and SWS (Carr et al., 2012; Oliva et al., 2018; Figure 4). It should be noted that the increase in slow gamma coherence correlated with a more stable replay of place cells (memory consolidation) during run sessions in W-track (theta mode); however during rest sessions, this correlation was absent (Carr et al., 2012).

The occurrence of fast gamma (90–140 Hz) was also described during SWS (Csicsvari et al., 1999; Sullivan et al., 2011). Fast gamma and SWRs are linked and have many similar features. Like SWRs, fast gamma occurs in bursts from 40 to 100 ms. SWRs and fast gamma have a similar current source density profile (although not identical). The phase locking of pyramidal cells and interneurons to SWRs and fast gamma is strongly correlated. The amplitudes of SWRs and fast gamma bursts during recording from different sites also significantly correlate (Sullivan et al., 2011). The general pattern of coherence during fast gamma episodes is similar to that of SWRs; i.e., if two sites are coherent in SWRs frequencies, then they are also coherent in fast gamma (Sullivan et al., 2011). Despite the significant similarity, fast gamma and SWRs possess different spectral properties (the frequency peak is within 90–120 and 120–200 Hz, respectively). Fast gamma bursts have a smaller amplitude, in general 1.5 times lower than that of SWRs. The reason for the similarity of SWRs and fast gamma is probably that a signal arrives to the same neural ensembles whose activation we observe as high-frequency oscillations (Sullivan et al., 2011).

Thus, gamma oscillations appear to be a consistent feature of SWRs, although it is evident that the non-theta neuronal and network activity represents an operational state different from the theta state. But how does the transition between theta and non-theta hippocampal states occur? Specific mechanisms of this process remain to be elucidated. In the next section, we will summarize the available experimental data concerning the transition between hippocampal theta and non-theta states and propose possible mechanisms underlying this transition.

## 3. Transition between hippocampal theta and non-theta states

Transitions between different hippocampal functional states occur relatively fast, within a few seconds (Schultheiss et al., 2020). During behavioral state transitions, hippocampal oscillatory activity may drop in several frequency bands (Lapray et al., 2012). The synchrony in one frequency band is suddenly replaced by synchrony in another. Pronounced delta or theta rhythm usually occurs when synchrony in the other band is weak. However, it is worth noting that such transitions are not always “clean”: in some cases, delta and theta correlate positively over short time intervals (Schultheiss et al., 2020).

When the behavior of an animal changes from the theta to the non-theta state, the hippocampal network excitability also changes. The activity of local interneurons, as well as septal and entorhinal inputs has different temporal structure compared with theta oscillations. During transitions from the theta to the non-theta state in freely moving and sleeping rats, the firing rate of hippocampal theta cells (presumably interneurons) may be significantly reduced. In contrast, the activity of complex-spike cells (presumed pyramidal cells) may increase (Stewart, 1993). Recording hippocampal CA1 units in awake guinea pigs, Rivas and colleagues showed that, when the animals change from the standing position to walking, the majority of units (presumed pyramidal cells) increased their rhythmicity and/or firing frequency or phase-locking with theta rhythm (Rivas et al., 1996). More recently it was shown that these transitions between different behavioral states with overall low oscillatory activity are accompanied by a decrease in the firing of PV basket cells, which may facilitate the network reorganization (Lapray et al., 2012; Kocsis et al., 2022).

### 3.1 Role of the medial septum in the control of hippocampal non-theta states

A key structure in the control of hippocampal rhythmical modes is the medial septum (MS; Vinogradova, 1995; Vertes and Kocsis, 1997; Buzsáki, 2002; Colgin, 2013). The lesion or pharmacological inactivation of the MS abolishes the theta rhythm and leads to a pronounced non-theta activity in the hippocampus (Green and Arduini, 1954; Petsche and Stumpf, 1962; Mizumori et al., 1990; Vinogradova et al., 1993; Mitchell et al., 2008; Wang et al., 2015). Electrical, pharmacological, and optogenetic stimulation of the MS, on the contrary, induces a stable theta rhythm (Brazhnik et al., 1985; Vandecasteele et al., 2014; Astasheva et al., 2015).

The MS is composed of glutamatergic, cholinergic, and GABAergic neurons (Brashear et al., 1986; Kiss et al., 1997; Sotty et al., 2003; Table 1). The population of GABAergic neurons is heterogeneous and consists of calretinin- and PV-expressing cells. All neuronal populations, except calretinin-containing GABAergic cells, project to the hippocampus (Mesulam et al., 1983; Freund, 1989; Manseau et al., 2005). For more detail about MS neurons and their interactions, see the works of Mysin et al. (2015), Tsanov (2015), and Müller and Remy (2018).

Glutamatergic hippocampal MS projections innervate both pyramidal cells and interneurons (Manseau et al., 2005; Fuhrmann et al., 2015). There are conflicting data on their physiological role. On the one hand, glutamatergic MS neurons were found to excite directly interneurons that control the input to CA1 both from Schaffer collaterals and the perforant pathway (Fuhrmann et al., 2015). On the other hand, the optogenetic stimulation of MS glutamatergic projections in the fimbria-fornix had no effect on the hippocampal theta rhythm (Robinson et al., 2016). On the contrary, the stimulation of glutamatergic cell bodies inside the MS induced the hippocampal theta rhythm whose frequency was strictly modulated by stimulation (Fuhrmann et al., 2015; Robinson et al., 2016). Thus, intraseptal glutamatergic connections seem to be much more important for the regulation of hippocampal rhythmical modes. Direct MS glutamatergic projections to the hippocampus probably play only a modulating role and may take part in the formation of place cells (Fuhrmann et al., 2015).

The role of MS cholinergic neurons in the regulation of hippocampal rhythmical activity seems to be similar. The stimulation of cell bodies of MS cholinergic neurons generates the sustained theta rhythm in the hippocampus (Vandecasteele et al., 2014; Dannenberg et al., 2015). The stimulation of their axon terminals in the hippocampus, on the contrary, has only a modulating effect: an increase in the activity of interneurons and a decrease in the firing rate of pyramidal cells (Dannenberg et al., 2015). However, some authors believe that MS cholinergic neurons may *directly* affect the hippocampal activity, maintaining a high level of acetylcholine in the hippocampus (Ma et al., 2020). By using the long-term recording of MS cholinergic neurons and optogenetic methods it was shown in freely behaving mice that these neurons were highly active during theta-dominating states (active exploration and REM sleep). During non-theta states (more specifically, during SWS) MS cholinergic neurons were almost silent, and their optogenetic activation suppressed ripple oscillations in CA1 (Ma et al., 2020).

**Figure 5:**
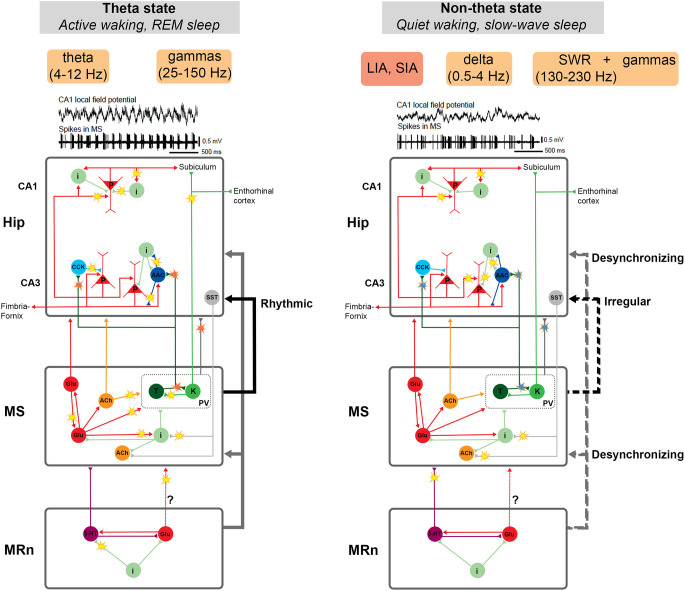
Schematic representation of neuronal circuits that participate in two main operational modes of the hippocampus: theta and non-theta state. The theta state is typical for active wakefulness and REM sleep. In this state, a stable theta rhythm and associated gamma rhythms of various frequencies are recorded in the hippocampus. The non-theta state manifests itself during quiet wakefulness and slow-wave sleep. It is characterized by irregular high-amplitude or low-amplitude delta activity in the hippocampus interrupted by sharp-wave ripple complexes (SWRs), which may be associated with gamma oscillations of various frequencies. The key structure in the regulation of hippocampal activity is the medial septum (MS). Disturbance of the balance of mutual inhibition between specific groups of MS cells (probably, Teevra cells suppress Komal cells) results in a more constant inhibitory signal from Teevra cells to the hippocampus. This, in turn, may lead to disinhibition of pyramidal neurons, promote their synchronization and the generation of SWRs. Thus, the restructuring of the functioning of the MS neural network and the change in the activity pattern of MS cells creates the conditions for the emergence of either theta- or non-theta state. Hippocampal-septal connections may also be involved in this process. The median raphe nucleus (MRn), a known desynchronizing agent of hippocampal activity, probably controls the hippocampal non-theta state through the influence on the MS or even directly. Neuronal activity associated with the transition between the theta and non-theta states is marked by “flashes”. Red flashes represent rhythmical signals, blue ones—decrease in the cell rhythmicity. See details in the text. Abbreviations: AAC, axo-axonal cells; ACh, cholinergic cells; CCK, cholecystokinin-containing cells; i, interneurons; glu, glutamatergic cells; K, Komal cells; LIA, large irregular activity; MRn, median raphe nucleus; MS, medial septum; p, pyramidal cells; PV, parvalbumin-expressing cells; REM, rapid eye movement sleep; SIA, small irregular activity; SST, somatostatin-positive neurons; SWRs, sharp wave-ripple oscillations; T, Teevra cells; 5-HT, serotonergic cells. Examples of LFPs and unit firing are adapted with permission from Bragin et al. (1995).

PV GABAergic MS neurons seem to make the main contribution to the regulation of hippocampal functional states. It is GABAergic MS neuronal activity that correlates most strongly with the hippocampal theta rhythm (Borhegyi et al., 2004; Varga et al., 2008; Hangya et al., 2009). Experiments with the direct optogenetic activation of PV GABAergic MS neurons support this notion. The stimulation of both GABAergic MS cell bodies and hippocampal terminals induces the theta rhythm and impedes the non-theta activity (Figure 5). The oscillation frequency in hippocampal LFPs linearly depends on the frequency of stimulation (Dannenberg et al., 2015; Zutshi et al., 2018).

Projecting GABAergic MS neurons have been subdivided into two populations (Borhegyi et al., 2004; Henderson et al., 2004). The neurons of these populations were named Teevra and Komal cells due to peculiarities of their spike train dynamics (Joshi et al., 2017; Viney et al., 2018). Teevra cells have a short burst duration (“sharp sound of burst”) and Komal cells have a long burst duration (“soft sound of burst”). Teevra and Komal cells suppress each other and discharge in antiphase bursts (Joshi et al., 2017). Teevra and Komal cells have many significant differences. Their cell bodies are located in different parts of the MS, closer to the center or the periphery respectively (Henderson et al., 2004). Teevra cells are the most rhythmic among MS neurons and fire preferentially at the troughs of hippocampal LFPs. Komal cells discharge mainly at the peak of CA1 theta LFPs (Borhegyi et al., 2004; Joshi et al., 2017). Teevra cells preferentially innervate CCK basket and axo-axonal neurons of the CA3 field (Joshi et al., 2017), while Komal cells send projections to the subiculum and the entorhinal cortex (Viney et al., 2018).

Computational experiments show that the antiphase discharge regime of two populations of mutually inhibitory bursting neurons is very unstable. Small deviations in the mutual inhibitory balance lead to the complete suppression of one population by the other (Mysin et al., 2015). The stability of the antiphase regime is most likely provided by glutamatergic and cholinergic neurons of the MS. A computational model was developed that reproduces this effect (Mysin et al., 2015). Thus, although glutamatergic and cholinergic MS neurons have direct hippocampal projects, their role is not clear. Local MS connections are very important for maintaining the activity of Teevra and Komal cells (Wu et al., 2003; Dannenberg et al., 2015; Robinson et al., 2016).

Despite numerous data on the control of the theta rhythm by MS projections, the role of the MS in controlling the non-theta hippocampal activity has not been adequately studied. On the one hand, complete suppression of MS neuronal activity by procaine or muscimol leads to the non-theta state in the hippocampus, very similar to quiet wakefulness or non-REM sleep (Smythe et al., 1991; Lawson and Bland, 1993; Vinogradova et al., 1993; Wang et al., 2015). Based on these data, it could be assumed that the non-theta state in the hippocampus arises as a result of the lack of input from the MS.

However, recent data on the peculiarities of hippocampal innervation allow us to hypothesize that the MS plays a more active role in the hippocampal non-theta state. PV GABAergic MS neurons are not silent in non-theta state. Experiments on unanesthetized head-fixed mice show that there is no significant difference in their firing rate between theta and non-theta states (Simon et al., 2006). In a recent study, when a much larger number of neurons is considered, a certain decrease in their firing rate in the non-theta is observed (Kocsis et al., 2022). Experiments on anesthetized animals show a more substantial decrease in firing rate in the non-theta state, but even in this state PV GABAergic MS neurons discharge with non-zero frequency (Borhegyi et al., 2004; Kocsis et al., 2022). It is interesting that the firing pattern of PV GABAergic MS neurons is likely dependent on the cell population. Teevra cells maintain their firing rate (Joshi et al., 2017), while Komal cells reduce their firing rate compared to the theta state (Unal et al., 2015; Joshi et al., 2017; Viney et al., 2018). At the same time, all subpopulations of PV GABAergic MS neurons decrease their rhythmicity in the absence of hippocampal theta rhythm (Joshi et al., 2017; Viney et al., 2018; Kocsis et al., 2022). Based on these data, we assume that in the non-theta state, the antiphase mode of discharge of Teevra and Komal cells is destroyed; i.e., Teevra cells suppress Komal cells. This, in turn, results in a more constant inhibitory signal from Teevra cells to the hippocampus. A more stable inhibition of CCK-positive and axo-axonal neurons reduces the perisomatic inhibition of pyramidal neurons. The disinhibition of pyramidal cell bodies allows them to discharge with a higher probability, facilitating the generation of complex spikes and ripple oscillations as a process of synchronization of pyramidal neurons in the CA3 due to local excitatory connections (Viney et al., 2013; Buzsáki, 2015).

The possible mechanism of SWR initiation through the disinhibition of pyramidal cells has experimental confirmations. Axo-axonal interneurons in freely moving rats do not fire during SWR (Viney et al., 2013). At the same time, according to some data, Teevra cells do not change their firing during SWR (Joshi et al., 2017). But in an earlier study, it was shown that around 40% of presumably Teevra cells were activated during hippocampal SWR (Viney et al., 2013). Probably, local interneuronal connections also significantly contribute to the suppression of axo-axonal cells. Axo-axonal cells inhibit other interneurons and receive inhibitory responses (Rees et al., 2016). A stable suppression of axo-axonal neurons by Teevra cells may provoke a weakening of their ability to compete with other populations of interneurons. The functional reorganization of the MS neural network and, as a result, a change in the firing pattern of Teevra cells creates conditions for theta or non-theta hippocampal states (Figure 5).

Interactions between the MS and the hippocampus are reciprocal and depend on the state of the network. Thus, hippocampal projections to the MS are also likely to be involved in the regulation of the neuronal network states. Hippocampo-septal communication is mediated by somatostatin-positive CA1-CA3 neurons (Toth et al., 1993; Jinno and Kosaka, 2002). These projecting neurons receive input from pyramidal neurons and granule cells and are involved in rhythmic processes in the hippocampus (Blasco-Ibanez and Freund, 1995; Gulyás et al., 2003; Takács et al., 2008). High- and low-frequency stimulations of hippocampo-septal neurons have different effects. Using the optogenetic approach, Mattis and colleagues stimulated hippocampo-septal afferents in an acute slice preparation (Mattis et al., 2014). Stimulations at both theta and ripple frequencies elicited a fast GABAergic postsynaptic response. However, in response to prolonged high-frequency (ripple) stimulation, a slow hyperpolarization of cholinergic and GABAergic MS neurons was observed (Mattis et al., 2014). It was shown in behaving rats that 60% of MS cells were significantly suppressed during SWR (Dragoi et al., 1999). Thus, periodically occurring ripple events may support the functioning of the MS neural network in the non-theta mode.

The role of the rhythmicity of MS input in the formation of hippocampal theta and non-theta states was confirmed in model studies (Tokuda et al., 2019). Transitions between the theta and non-theta states were investigated using a dynamic model of the local hippocampal circuit. In the model, as well as in the real hippocampus, neurons demonstrated highly synchronous periodic oscillations in the presence of rhythmic input from the MS. Aperiodic MS activity led to diffusion-induced chaotic dynamics, just as the disappearance of the rhythmicity of MS neurons led to LIA in place of theta in the real hippocampus (Tokuda et al., 2019).

In conclusion, we should note that the role of the MS in maintaining the hippocampal non-theta state is not completely clear and needs to be proven more directly.

### 3.2 Role of the median raphe nucleus in the control of hippocampal non-theta states

A considerable body of evidence indicates that other brain structures are also involved in the generation of, and transition between hippocampal functional states. The median raphe nucleus (MRn), a known desynchronizing agent of hippocampal activity, probably controls the hippocampal non-theta state through the influence on the MS or even directly (Vertes, 1981; Vertes and Kocsis, 1997; Kitchigina et al., 1999; Vinogradova et al., 1999; Figure 5). It was shown in awake rabbits that low-amplitude MRn stimulation disrupted MS bursting and abolished theta activity in the hippocampus, while the suppression of MRn activity by local injections of lidocaine had the opposite effect (Kitchigina et al., 1999; Vinogradova et al., 1999). It is thought that serotonergic MRn neurons may favor the hippocampal non-theta states, suppressing theta synchronization in the MS and/or hippocampus (Vertes and Kocsis, 1997; Varga et al., 2002; Kudina et al., 2004; Vertes, 2010).

Different functional modes of the hippocampus may also be supported by other neuronal pathways and neurotransmitter systems in the MRn-MS-hippocampus circuit. Thus, it was shown that non-serotonergic (possibly glutamatergic) projections of the MRn to the MS contribute to the generation of the theta state in anesthetized rats in the absence of MS cholinergic tone (Crooks et al., 2012). Earlier, an elevated level of glutamate in the MRn was found to coincide with irregular desynchronized hippocampal activity (non-theta state; Varga et al., 1998). The authors suggested that theta-related changes in the MRn glutamate level may reveal downstream forebrain influence and represent a feedback regulation of hippocampal activity (Varga et al., 1998).

Interestingly, Jackson and colleagues demonstrated that high-amplitude MRn stimulation provoked SIA in the hippocampus rather than other “typical” non-theta activities like LIA, SWR or slow oscillations (Jackson et al., 2008). Thus, the desynchronizing action of MRn caused a marked general reduction in the amplitude of hippocampal LFPs and a deep blockade of theta. The stimulation of MRn failed to affect theta-unrelated hippocampal cells but suppressed the activity of the majority of both theta-on and theta-off cells (Jackson et al., 2008). On the contrary, theta-off cells were observed to discharge continuously during LIA (Colom and Bland, 1987; Ford et al., 1989). Although the response to MRn stimulation was dependent on the brain state, cell type, and theta phase, the authors supposed that MRn stimulation could elicit a hippocampal non-theta state dissimilar to LIA (Jackson et al., 2008).

It could be supposed that the suppression of theta state by serotonergic projections of the MRn may indicate that the transition between hippocampal theta and non-theta states is an active process.

## 4. Functional significance of hippocampal non-theta states

Cortical slow and delta oscillations are believed to play an important role in memory consolidation, especially by facilitating the information flow between the hippocampus and the neocortex (Sirota et al., 2003; Maingret et al., 2016; Todorova and Zugaro, 2019). The functional significance of hippocampal delta oscillations and non-theta activity in general is not as well characterized. It has been noted in several studies that learning during non-theta states is less effective than during theta states. In the absence of theta oscillations, the rate of acquisition of eyeblink conditioning in rabbits was significantly slower, and the percentage of conditioned responses was lower compared with animals trained during theta states (Seager et al., 2002; Griffin et al., 2004; Hoffmann and Berry, 2009; Cicchese and Berry, 2016).

**Figure 6:**
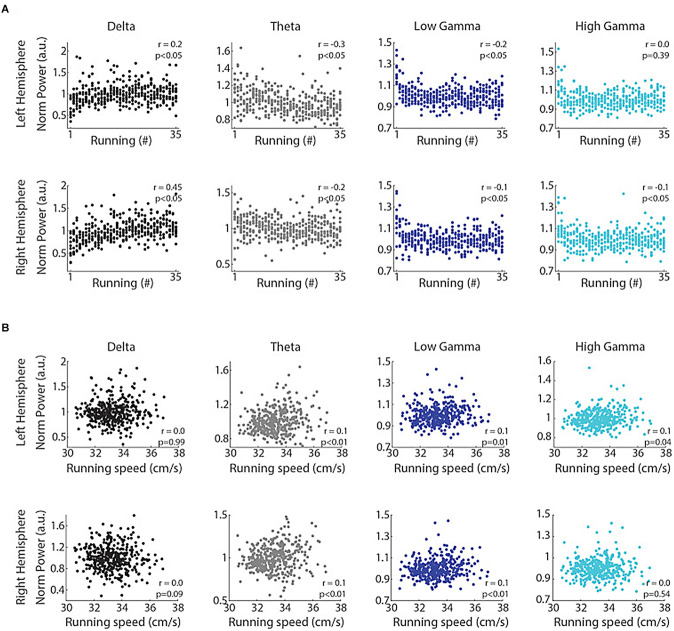
Consecutive runs promote specific increases in the power of delta oscillations in the rat hippocampus. Delta power correlates with the trial number but not with running speed. **(A)** Normalized delta band power during treadmill running and the trial number (“running #”) directly correlated (*r* = 0.2, *p* < 0.05 and *r* = 0.45, *p* < 0.05, for left and right hemispheres, Spearman’s correlation), while the power of theta, low gamma and high gamma bands inversely correlated with the trial number (*p* < 0.05, Spearman’s correlation), except high gamma power in the left hemisphere (*p* = 0.39). **(B)** Normalized delta band power and running speed were not correlated (*p* = 0.99 and *p* = 0.09 for left and right hemispheres, respectively, Pearson’s correlation), while the power of theta, low gamma and high gamma bands weakly and positively correlated with running speed (*p* < 0.05, Pearson’s correlation), except high gamma power in the right hemisphere (*p* = 0.54). Reproduced with permission from Furtunato et al. (2020).

The traditional view that theta and gamma represent the “online”, and delta reflects the “offline” mode of information processing, is opposed by the results of Furtunato and colleagues (Furtunato et al., 2020). Analyzing LFPs in the left and right rat hippocampi during consecutive short-term treadmill runs at the same speed, they found prominent delta and theta oscillations. Delta power and interhemispheric phase coherence in the delta band increased during consecutive treadmill runs. At the same time, theta and gamma power decreased with the trial number (Figure 6). Thus, consecutive treadmill runs enhanced delta power and inter-hemispheric phase coherence in the rat hippocampus (Furtunato et al., 2020).

In general, it is believed that, in rodents, theta and non-theta hippocampal modes reflect different network coupling and distinct behavior. Analyzing the cortical-cortical and cortical-hippocampal coherence in freely behaving rats, Young and McNaughton showed that the intensity of theta and delta oscillations clearly depended on animal behavior (Young and McNaughton, 2009). As opposed to theta activity, the power of the delta rhythm was maximal during grooming and resting, and minimal during locomotion, exploratory behavior, and rearing. During “non-cognitive” behavior, there was also an extensive delta coupling in the frontal cortex, which was not associated with the posterior areas and the hippocampus (Young and McNaughton, 2009). The authors believed that rhythmic activity of different frequencies may bind different neuronal ensembles into functional groups in different circumstances. In a recent study on freely behaving rats, this assumption received confirmation (Schultheiss et al., 2020). The periods of delta-dominated activity were shown to be prominent in the hippocampus when animals are motionless or moving slowly (non-theta state). Such delta-dominated modes were orthogonal to theta modes prevailing in running. Delta power and synchrony in the hippocampus were negatively related to the speed of an animal. The orthogonality of delta and theta modes was also manifested in the interaction of the hippocampus with medial PFC. While theta coherence between these structures increased during running, delta coherence increased during quiet wakefulness (Schultheiss et al., 2020).

Thus, the hippocampal delta rhythm, along with theta, may serve as a means of communication between the hippocampus and the neocortex (Roy et al., 2017; Mofleh and Kocsis, 2021). Roy and coauthors observed decreased delta coherence between the hippocampus and the PFC in anesthetized animals when the influence of the nucleus reuniens of the thalamus was abolished (Roy et al., 2017). The authors suggested that rhythmic couplings in delta and theta bands may serve as parallel communication channels between the PFC and the hippocampus operating in opposite directions. Later, the coupling between the hippocampus and the PFC at the respiratory rate (2 Hz, delta-range) was also shown in freely behaving rats (Mofleh and Kocsis, 2021).

To provide a more complete description of cognitive processes occurring in the non-theta state, we will briefly review the functions of SWRs. The most discussed functions of SWRs are memory consolidation, route planning, and information transfer from the hippocampus to the neocortex. These functions are based on the phenomenon of reactivation of theta sequences, i.e., the activation of place cells in the same neuronal ensemble in the order in which the rat traverses place fields (Both et al., 2008; Buzsáki, 2015). The hypothesis about the role of SWRs in memory consolidation is based on the fact that the SWRs-related reactivation of place cell sequences is accelerated compared to the theta-state reactivation. The activation of place cells in the “correct” order with an interval of a few milliseconds should strengthen the connection between neurons according to the STDP (Bi and Poo, 1999). This hypothesis received direct experimental confirmation. The blockade of SWRs generation by commissural stimulation impairs memory consolidation (Girardeau et al., 2009). There is also a lot of indirect evidence. For example, it was shown that the stability of the reactivations of pyramidal cells positively correlates with the quality of memory consolidation (Carr et al., 2011).

The hypothesis about the “route planning” (retrieval of “episodic-like” memory) function of SWRs is based on observations that place cells are activated in the order in which the animals later will run through the maze (Pfeiffer and Foster, 2015; Wu et al., 2017; Pfeiffer, 2020). Studies in humans also show that SWRs play a role in episodic memory retrieval. Sakon and Kahana found that just before word recall from previously studied lists, rate and power of SWRs raised. Moreover, if the words in the sequence were semantically related (for example, fish and whale, cupcake and pie), then the effect was stronger (Sakon and Kahana, 2022).

In 1971, D. Marr proposed the idea of the hippocampus as a temporary storage of information (Marr, 1971; Willshaw and Buckingham, 1990). This hypothesis received many confirmations, and SWRs are considered today as a reflection of the information transmission from the hippocampus to the neocortex (Willshaw and Buckingham, 1990; Buzsáki, 2015). The main arguments in favor of this hypothesis are the data on the induction of neuronal and field activity in the neocortex after SWRs in the hippocampus during SWS. Hippocampal SWRs were shown to induce delta oscillations and sleep spindles in the PFC (Sirota et al., 2003; Maingret et al., 2016; Todorova and Zugaro, 2020; Skelin et al., 2021). Other studies demonstrate the reactivation of neuronal ensembles in the PFC after hippocampal SWRs (Peyrache et al., 2009), as well as the important role of the hippocampus in stabilizing neuronal patterns in the PFC after sleep (Euston et al., 2007). In the context of this hypothesis, it is important to understand how cognitive processes occurring during hippocampal delta oscillations are related to SWRs. We have already noted that the hippocampal delta rhythm may also serve to interact with the neocortex. However, it is not known whether there is any connection between cognitive processes occurring in the non-theta state “outside” and “inside” SWRs.

Whether all described functions “coexist” in one SWR event is unknown. Perhaps SWRs are heterogeneous, and some cognitive operations are performed in one SWR, and others in another (Buzsáki, 2015). But it is evident that SWRs are the most discussed aspect of the non-theta state. On the other hand, there is no scientific consensus about the cognitive load of the non-theta-non-SWR mode.

## 5. Peculiarities of non-theta states in humans

It is common practice that patients suffering from drug-resistant epilepsy undergo invasive electrophysiological diagnostics with implantation of intracranial depth electrodes to localize the focus of seizure onset. This provides a unique opportunity to conduct additional studies of the rhythmic activity of the hippocampus in humans.

When recording hippocampal activity in 10 patients, Watrous and colleagues attempted to determine the behavioral correlates of human delta and theta rhythms. They showed that the power of delta and theta activity increased with increasing speed of navigation in virtual reality on a significantly larger number of electrodes than would be expected if it happened randomly. However, delta and theta power were modulated more uniformly by the “spatial view” when subjects looked at “spatial landmarks” in the virtual environment (Watrous et al., 2011). Later, the same group of researchers demonstrated that low-frequency oscillations (delta and theta) in the human hippocampus are not associated with sensorimotor processing but are involved in encoding the spatial information, for example, distance-related (Vass et al., 2016). This was shown with the help of short- and long-distance teleporters in virtual reality. Navigating in a cross-shaped environment, patients could use short-distance teleporters, placed about half way along the plus maze arms, or long-distance teleporters, placed near the ends of the maze arms. By the locations of teleporters, patients could predict the distance they would travel (short or long). The use of teleporters made it possible to avoid sensorimotor processing during navigation (visual cues were absent). During long-distance teleportation (the teleportation time did not depend on the distance), the periods of low-frequency oscillations were also longer. Thus, the pattern of oscillations in the hippocampus during teleportation made it possible to determine what the teleported distance was, long or short (Vass et al., 2016). It is worth to note, that, studying neuronal activity in humans during virtual navigation, Jacobs and colleagues show that most hippocampal neurons are modulated by delta (1–4 Hz) rather than theta rhythm (4–8 Hz; Jacobs et al., 2007).

**Figure 7:**
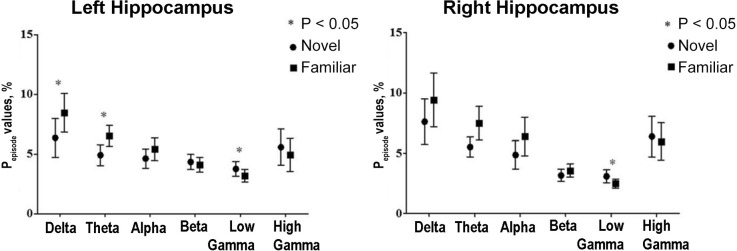
Human hippocampal delta and theta rhythms show a similar pattern of changes during navigation. The relation between hippocampal oscillations and memory encoding of environmental novelty in epileptic patients during spatial navigation. The total amount of time that the power of a specific frequency band is above a certain threshold for at least three cycles divided by the total amount of time in the block of the navigation task (*P_episode_* values, %) is presented for the first block (novel environmental condition) and the eighth (final) block (familiar environmental condition). Theta and delta oscillations significantly increase, but low-gamma oscillations decrease as the block progresses and the environment is being encoded. Reproduced with permission from Park et al. (2014).

Human hippocampal delta and theta rhythms (as well as gamma) may also be involved in encoding the environmental novelty. During the search for objects and memorizing their location in virtual reality, hippocampal delta and theta oscillations in the first block of tasks were smaller than in the subsequent ones, in contrast to the gamma rhythm, which was higher in the new environment (the first block of tasks; Park et al., 2014; Figure 7).

The synchrony of the rhythmic activity of the hippocampus and the entorhinal cortex was analyzed using recordings from the non-epileptic brain hemisphere of 22 patients with unilateral hippocampal sclerosis (Mormann et al., 2008). The analysis of phase coherence throughout different frequency ranges revealed a lower interstructural (entorhinal-hippocampal) synchrony in comparison with the levels of intrastructural synchrony. However, the effect reached statistical significance only in the delta and theta ranges. The authors suggested the presence of independent delta/theta rhythms in the entorhinal cortex and the hippocampus in humans (Mormann et al., 2008). It is interesting to mention here an earlier work in which it was shown that in patients with temporal lobe epilepsy theta coherence between the hippocampus and the perirhinal/entorhinal cortex increased with successful word memorization (Fell et al., 2003). Thus, the formation of declarative memory probably requires an active synchronization of independent delta/theta oscillations in the hippocampus and the entorhinal cortex.

In contrast to rodents, where the theta and delta modes probably reflect different brain states, in humans, a similar pattern of changes in hippocampal theta and delta oscillations is observed during navigation. Moreover, in humans, correlated change in the delta and theta rhythms is observed not only in the hippocampus, but also in the neocortex in virtual navigation and word memorization tasks (Kahana et al., 1999; Serruya et al., 2014). It can be assumed that the division of human hippocampal low-frequency activity into delta and theta bands is probably not always justified. Moreover, canonical frequency bands were determined on the basis of human *scalp* EEG studies (Niedermeyer, 1999) and may not accurately reflect the dynamics of oscillations in the hippocampus. It is suggested in the literature that the human hippocampal theta range may extend from 1 to 8 Hz (Jacobs et al., 2007; Herweg and Kahana, 2018).

## 6. Conclusions

Non-theta and especially non-theta-non-SWRs hippocampal modes have been little studied. In most works, these states are defined as the absence of the theta rhythm, and are not based on the analysis of specific parameters. Although the frequency patterns of the non-theta state and its behavioral correlates (SWS, quiet wakefulness) are known, many parameters of hippocampal neuronal activity, as well as causal relationships between neuronal and field activities, remain to be clarified. Based on the reviewed data, we provide a list of the most important unresolved questions about the non-theta state:

• Is the non-theta state homogeneous? Are there only two operational modes in the hippocampus?
• What is the mechanism of delta rhythm generation? What groups of neurons are involved in it?
• Does information processing take place in the non-theta-non-SWRs state? Is non-theta-non-SWRs neuronal activity the result of information processing or is it just a noise? What methods can be used to measure information processing in the non-theta-non-SWRs state?
• What causes the difference in delta and theta rhythms in humans and rodents? Are the delta frequency ranges the same in humans and rodents?

More extensive experimental and theoretical studies are needed to clarify these issues. The answers to these questions will bring us closer to understanding what the brain does when it rests and whether it rests at all.

## Author contributions

IM has made general structure of the article, abstract, introduction, conclusions and section “2. Hippocampal activity in the non-theta state”. LS has made figures and the following sections: “3. Transition between hippocampal theta and non-theta states”, “4. Functional significance of hippocampal non-theta states”, “5. Peculiarities of non-theta states in humans”. All authors contributed to the article and approved the submitted version.

## Abbreviations

CCK: cholecystokinin-containing cells
LFPs: local field potentials
LIA: large irregular activity
MRn: median raphe nucleus
MS: medial septum
OLM: oriens lacunosum moleculare cells
PfC: prefrontal cortex
PV: parvalbumin-expressing cells
REM: rapid eye movement sleep
SIA: small irregular activity
STDP: spike-timing-dependent plasticity
SWRs: sharp wave-ripple oscillations
SWS: slow-wave sleep.
